# Exploring the Potential of Pectin as a Source of Biopolymers for Active and Intelligent Packaging: A Review

**DOI:** 10.3390/polym16192783

**Published:** 2024-09-30

**Authors:** Andi Dirpan, Yosini Deliana, Andi Fadiah Ainani, Irwan, Nur Alim Bahmid

**Affiliations:** 1 Department of Agricultural Technology, Hasanuddin University, Makassar 90245, Indonesia; 2 Research Group for Post-Harvest Technology and Biotechnology, Makassar 90245, Indonesia; 3 Agribusiness Department, Faculty of Agriculture, Universitas Padjadjaran, Bandung 40161, Indonesia; 4 Food Crop Production Technology, Hasanuddin University, Makassar 90245, Indonesia; 5 Research Center for Food Technology and Processing, National Research and Innovation Agency (BRIN), Yogyakarta 55961, Indonesia

**Keywords:** bioactive compounds, bibliometric analysis, pectin, properties, bioplastics

## Abstract

The use of fossil-based plastics in food packaging poses a serious environmental concern. Pectin, a natural biodegradable polymer, offers a potential solution for environmentally friendly and sustainable food packaging to replace fossil-based plastics. This article reviews the applications of pectin in active and intelligent packaging and analyzes the latest research trends. Bibliometric analysis was used to review the existing literature on pectin in food packaging. Data were collected from the Scopus database, which covers research on film manufacturing and pectin-based coating. Pectin-based active packaging contains antimicrobial and antioxidant compounds such as ascorbic acid and essential oils, which effectively prevent bacterial growth while absorbing oxygen and water vapor. In contrast, pectin-based intelligent packaging allows real-time monitoring of food quality through integrated color-changing indicators, eliminating the need for open packaging. Research trends have shown a significant increase in publications on pectin-based packaging, reflecting the growing interest in sustainable packaging solutions. With a focus on innovation and sustainability, pectin can replace conventional plastics and provide safer and more durable packaging solutions, thereby supporting global efforts to reduce the environmental impact of plastic waste.

## 1. Introduction

Packaging plays an important role in the food industry by protecting food products from damage, contamination, and deterioration during distribution and storage. However, conventional packaging from petroleum-based polymers causes severe environmental problems owing to their non-biodegradable environmental properties. Since the beginning of the 21st century, fossil-based global plastic production has doubled, reaching 394 million tons in 2021 and continuing to rise to 400.3 million tons in 2022 [1]. Projections indicate that by 2060, the amount of plastic leaking into aquatic environments such as rivers, lakes, and oceans is expected to increase by 91%, reaching 11.6 million tons per year [2]. The accumulation of plastic waste originating from land and flowing into the oceans is anticipated to continue rising alongside rapid population growth [3]. Therefore, biodegradable packaging can be an alternative to conventional plastics.

Pectin is a natural polymer that shows promise as an ingredient for the manufacture of biodegradable plastics. Pectin is a natural polysaccharide found in plant cell walls that contains linear chains of D-galacturonic acid in α(1–4) bonds with some -COOH in the form of methyl esters [4]. Pectin can be extracted from a variety of natural sources, including fruits, such as oranges, apples, lemons, and grains. It can be derived from agricultural waste, such as lemon peel, pitaya, tomato, pomelo, dragon fruit, and passion fruit [5,6,7,8]. Pectin can be classified into two types: high-methoxyl pectin with a carboxyl esterification degree higher than 50% and low-methoxyl pectin with a carboxyl esterification degree less than 50% [9]. However, the commonly used pectin is high-methoxyl pectin. Pectin has attracted widespread attention as a potential source of biopolymers in food packaging because of its unique properties such as biodegradability [10], biocompatibility, and the ability to form a solid film [11]. In addition, pectin-based packaging is generally considered safe (GRAS) by the Food and Drug Administration (FDA) [12].

**Figure 1:**
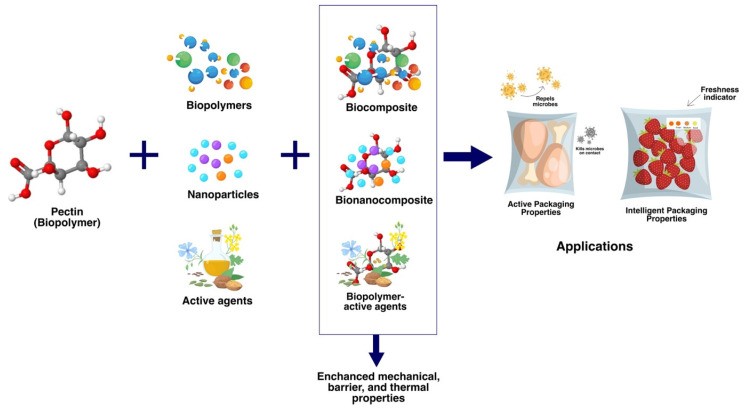
An overview of biopolymer-based pectin in active and intelligent packaging applications.

The development of food packaging films using bio-based biodegradable polymers, such as pectin, combined with natural or synthetic additives to improve material properties and product shelf life has recently become an attractive solution in the food industry. Active packaging contains active compounds with antimicrobial and antioxidant properties that extend the shelf life of food products by releasing active compounds that can prevent the growth of bacteria and absorb oxygen and water vapor in the package [13,14,15,16,17,18]. Many studies have added reinforcing agents, such as nanofillers [19,20,21,22], biopolymers [23,24,25], plasticizers, and natural substances, such as essential oils, into the packaging matrix [26,27]. Pectin-based films in active food packaging can slow fat transfer, such as the migration of fat from fatty foods to packaging [28], and help retain food moisture. In addition, intelligent packaging is often applied to biopolymer-based packaging because it allows consumers to assess and monitor food quality without damaging the packaging materials. Pectin-based films have also shown advantages in mechanical properties and their ability to act as barriers to aroma, oxygen, and water transfer, comparable to synthetic polymers in food packaging [29]. An overview of pectin as a biopolymer in food packaging is shown in Figure 1.

Many reviews on pectin, its structure, and extraction methods have been published. The structural changes, mechanisms, and applications of modified pectin have been previously reviewed [30]. Pectin modification methods have also been comprehensively discussed [31]. Researchers have also reviewed various conventional and non-conventional methods for extracting pectin from different sources and examined their bioactivity [32]. In addition, the potential of pectin to produce renewable and environmentally friendly packaging, in line with the concept of circular economy, has been evaluated [33] as well as its applications in various fields (food, pharmaceutical, and cosmetic industries) [34]. However, only a few reports have examined food packaging films using pectin as active and intelligent packaging, and none have comprehensively integrated the development trend of pectin as food packaging through a bibliometric approach. Therefore, this review aims to study the sources, extraction methods, physical properties, and applications of food packaging. It also comprehensively discusses the application of pectin-based active and intelligent packaging in food products. It also uses quantitative data and statistical analysis to assess the development and future direction of pectin-based food packaging research using a bibliometric analysis approach. This review is expected to provide insights to scientists and industry players regarding the potential of pectin as a natural polymer, its applications in active and intelligent food packaging, and the direction of its future development.

## 2. Systematic Review: Method and Outcome

### 2.1. Search Strategy

The search was conducted on 23 March 2024 using the Scopus database, which was chosen because it is widely recognized as a comprehensive source of publication data for systematic analysis and meta-analysis. The analysis was conducted based on the search query (TITLE-ABS-KEY ((“pectin*”) AND (film*) OR (“coating”) AND (“food packag*”)). Only articles published between 1998 and 2024, written in English, in the format of a review or article type of document from a journal source, and in the final publication stage were selected. Documents that did not meet these criteria were excluded. This rigorous selection process resulted in a total of 310 documents being downloaded and saved in CSV file format. These documents contained citation information (author, document type, year, etc.), bibliographical information, abstract, and keywords for further analysis. The documents were then converted into Microsoft Excel to revise erroneous keywords. Subsequently, to reduce bias, Openrefine was used to clean keywords with the same meaning but different forms of writing. The merged words biopolymer and biopolymers were merged into biopolymers; polyvinyl alcohol and poly (vinyl alcohol) were merged into polyvinyl alcohol; nanoemulsion and nanoemulsions were merged into nanoemulsion; Active food package and active food packaging were merged into active food packaging; mechanical properties and mechanical property were merged into mechanical properties; and pectin film and pectin films were merged into pectin films.

### 2.2. Data Analysis

This analysis covers a wide range of indicators, including the frequency, trends, rankings, network analysis, citations, and evaluation of word occurrences. For an additional in-depth analysis, applications such as Vosviewer v.1.6.19 and Tableau were used. These tools map co-occurrences with keywords, enabling the identification of key study areas, visualization of country maps, and detection of emerging research trends.

## 3. Sources and Characteristics of Pectin

Pectin is a complex polysaccharide naturally present in all plant cell walls and lamellae. Pectin can be extracted from fruits, vegetables, and other plants. However, the primary sources are citrus peels and apple peels/pulp because of their high extraction yield and availability as food processing industry waste [35,36]. Agricultural byproducts can also be new sources of pectin, such as banana peels [37], mango peels [38], pomelo peels [39,40], cacao waste [41,42], and coffee pulp and grounds [43,44]. Recent studies have shown some potential sources of pectin such as from sugar beet pulp, with the hot acid extraction method yielding 28% pectin [45], and the microwave-assisted method (MAE) reaching 37% [46]; from Jabuticaba peel yielding 22% pectin [47], and *Passiflora tripartita* peel extract yielding 23% [48]. Pectin-based composite films prepared with Schiff base (GS) compounds synthesized by γ-aminobutyric acid (GABA) showed potential applications in fruit preservation as packaging materials [49]. Pectin-based composite films incorporated with cannabidiol/2,6-di-O-methyl-β-cyclodextrin inclusion complexes for food packaging were also reported to have good performance in strawberry preservation [50]. In addition, modified pectin has broad potential in packaging applications and other sectors. Pectin modified with fatty acids exhibits improved hydrophobicity and moisture resistance, making it particularly suitable for biodegradable packaging [51]. Then, modification with phenolic acids provides antibacterial and antioxidant properties, making them effective for active packaging that extends the shelf life of food [52]. Furthermore, pectin modified with resorcinal and 4-hexylresorcinol also showed significant improvements in antioxidant and antibacterial properties, which are effective for extending the shelf life of meat, making it suitable for active packaging in the preservation of meat products [53].

The characteristics of pectin in commercial use are strongly influenced by the source of the pectin material, extraction method, residual galacturonic acid content, degree of methoxylation/esterification (DM/DE), neutral sugar composition, and molecular weight. The properties of pectin, such as its solubility, gelling ability, and film-forming ability, are highly dependent on the source and degree of esterification. Pectin with a high methoxyl (>7%) content has a DE > 50% and pectin with a low methoxyl (<7%) content has a DE < 50% [37]. Packaging films with a high DE tend to exhibit better gel strength, viscosity, and stability under different storage conditions [54]. Pectin films with a high degree of esterification also exhibit lower water absorption and better mechanical resistance, making them more suitable for food packaging applications that require an effective barrier to moisture and gases [55]. In addition, the addition of plasticizers such as glycerol can increase the flexibility of pectin film [56]. Thus, the degree of esterification of pectin affects the physical and mechanical properties of films for food packaging applications.

**Figure 2:**
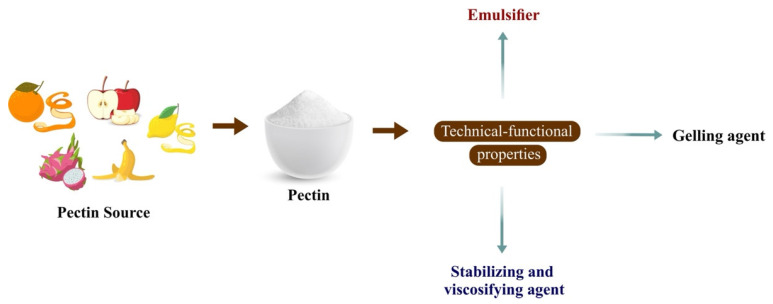
Techno-functional properties of pectin.

Pectin has several significant technical and functional properties (Figure 2). In the food industry, pectin is often used as a thickening and stabilizing agent. In addition, pectin has biodegradability [50], biocompatibility [57], and edibility properties [58], making it suitable as a polymeric matrix for manufacturing active edible packaging films [59]. Other studies have shown that blending pectin with other polymers, such as pullulan, can improve the properties of pectin films [60,61]. This combination forms intermolecular hydrogen bonds that improve the thermal stability and surface hydrophobicity of the film, which are particularly important for food packaging applications [60]. Pectin can also be incorporated into bioactive components to improve food product functionality. The development of pectin composite films with the addition of nanoparticles such as titanium oxide (TiO_2_) improves the mechanical and water vapor barrier properties. While pectin serves as the primary polymer providing film-forming capability, the addition of TiO_2_ specifically enhances these properties, including providing UV light filtering capacity, making these films ideal environmentally friendly and functional food packaging materials [62]. Additionally, pectin films can be modified with other natural ingredients to enhance their antimicrobial and antioxidant properties. Although pectin itself does not possess significant antimicrobial and antioxidant capabilities, it serves as the primary polymer, forming films with good mechanical properties, low water vapor permeability, and the unique ability to bind and release antimicrobial and antioxidant compounds in a controlled manner [59,63]. For example, the addition of polyphenol extracts from tea to pectin films can enhance their antioxidant and antimicrobial activities, which are crucial for extending the shelf life of food products [64]. Based on this, pectin offers an innovative and environmentally friendly solution for food packaging applications, providing effective protection against microbes and oxidation and improving the quality and safety of food products.

## 4. Bibliometric Analysis

### 4.1. Trend of Publication

**Figure 3:**
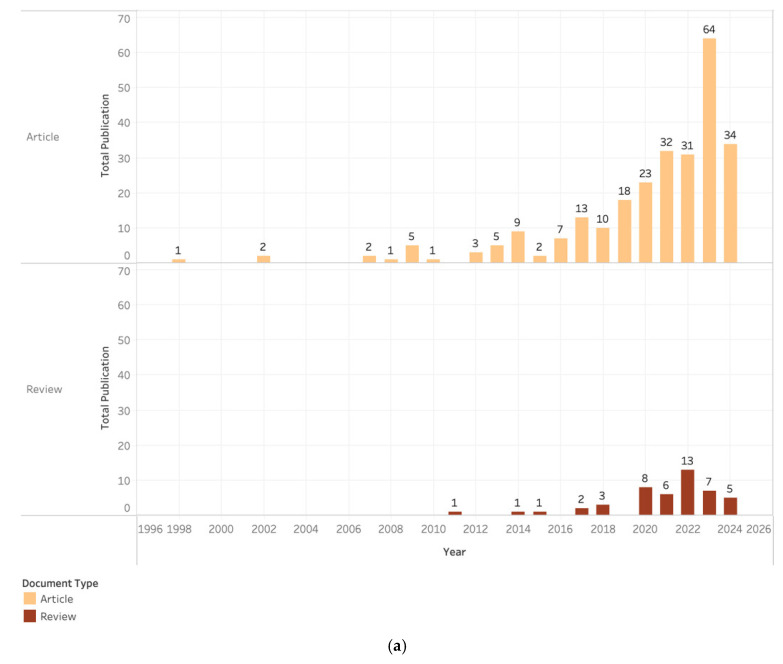

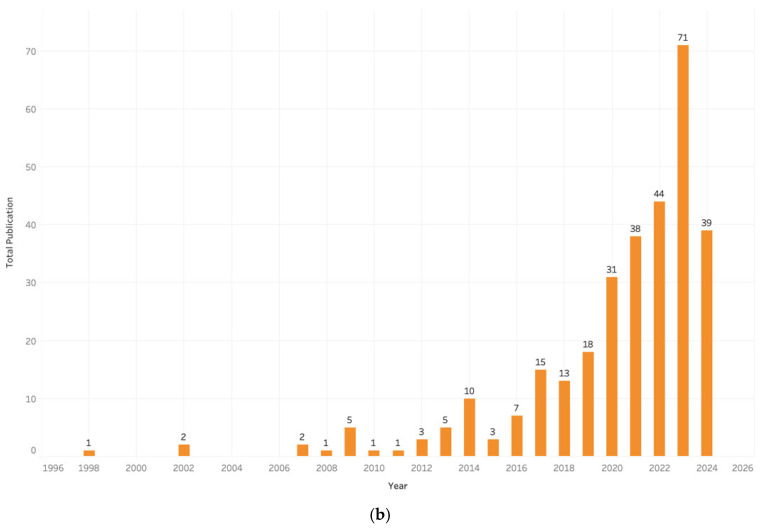
Annual production of pectin-based food packaging publications: (**a**) document type; (**b**) total publications.

Figure 3 illustrates the annual publication pattern (1998–2024) on using pectin as a food packaging material. Figure 3a shows the number of documents obtained based on the types of articles and reviews; 263 documents were articles (84.83% of the total documents) and 47 documents were reviews (15.16%).

The development of publications was divided into three stages: budding, development, and explosion (Figure 3b). The budding period, which lasted from 1998 to 2015, was characterized by a gradual increase in the number of scientific articles focusing on pectin-based food packaging research. In 1998, a group of academics began to realize the importance of carbon neutrality and regularly conducted research on this subject. This phenomenon persisted until 2015. However, this period was characterized by slow progress in research related to pectin-based food packaging, as using fossil-based plastics is still an option in food packaging production. In addition, pectin is inadequate in producing characteristics resembling synthetic packaging. However, the few studies conducted during this period formed a strong foundation for future research on pectin-based food packaging.

The second stage (development period), which runs from 2016 to 2019, is characterized by consistent progress in pectin-based food packaging research papers. The gradual increase in publications is due to bio-based plastics having unique advantages over conventional plastics in reducing dependence on finite fossil resources and reducing greenhouse gas emissions.

The current usage of bioplastics is minimal, accounting for less than one percent of the total annual plastic production, which exceeds 390 million tons. Nevertheless, the market for bioplastics is expanding dynamically due to rising demand and the emergence of advanced materials, applications, and products. Furthermore, the growth of publications during this period can also be attributed to the existing tendency to utilize waste generated from agricultural and industrial conversions as a means of reducing environmental damage or utilizing substantial biomass resources for the production of high-value products, such as pectin. This period lays the groundwork for the potential future exponential growth in pectin-based food packaging research.

The third phase (explosion period), which runs from 2020 to 2024 (ongoing), is characterized by a significant surge in the number of pectin-based food packaging research articles. The increase in publications during this period is attributed to the rapid growth and innovation within the bioplastics industry. This industry has the potential to separate economic growth from resource depletion and environmental impact. Furthermore, the European Commission has acknowledged the significance of bioplastics in the bioeconomy and their ability to hasten the transition to a circular economy. The European Bioplastics Association, which represents the interests of the bioplastics industry in Europe, is collaborating closely with European institutions and other relevant stakeholders to shape a favorable economic and policy environment in Europe that will support the flourishing of the bioplastics industry. This drives the interest of scientists to continue developing bioplastic packaging with good properties and characteristics. It is important to note that this study does not include all articles produced in 2024, as the data for this year are still ongoing and are predicted to continue to increase until the end of the year.

### 4.2. Research Hotspot Trends Based on Keywords

**Figure 4:**
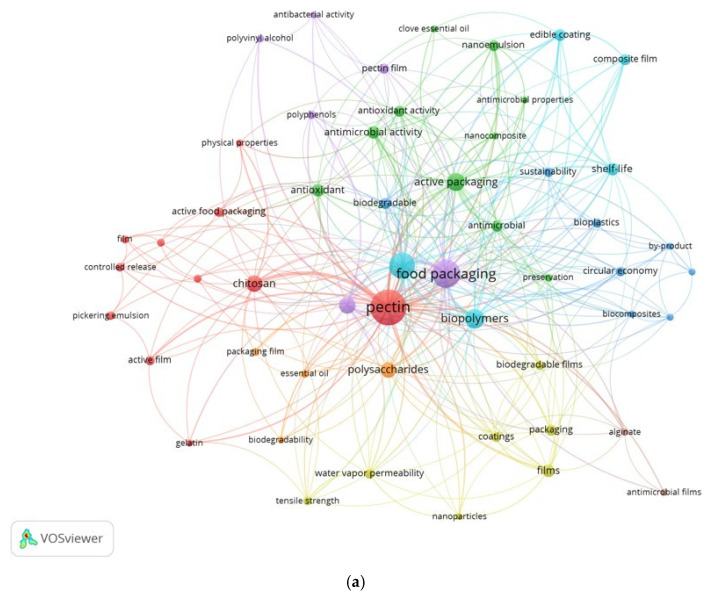

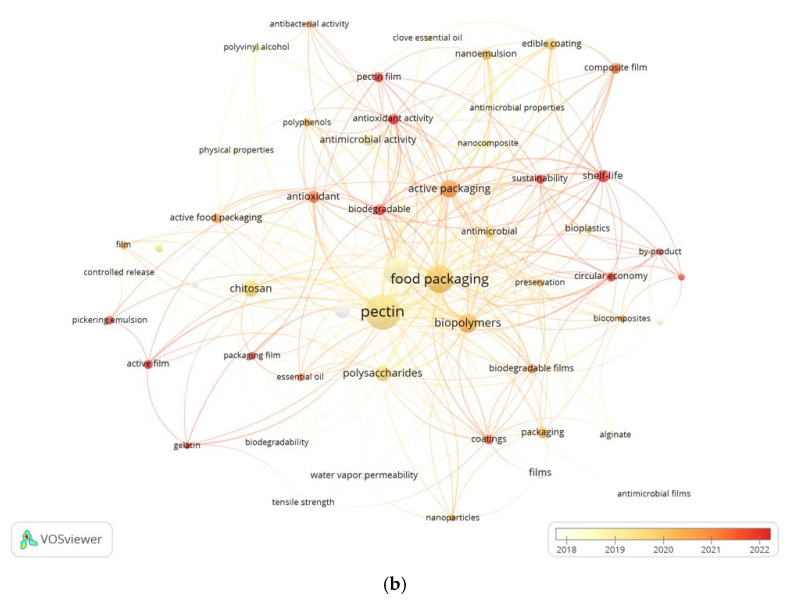
Co-occurrence network of 53 author keywords that appear at least four times. (**a**) Keyword network colored according to the generated clusters; (**b**) keyword co-occurrence period.

A bibliometric analysis was conducted to investigate current research trends in the utilization of pectin as a food packaging material. Using data from relevant scientific articles from Scopus, the analysis was conducted using VOSviewer to illustrate the occurrence of keywords. Of the 814 keywords collected, 53 keywords that appeared at least four times were selected for inclusion in the analysis. Several studies have shown that keyword analysis is an important component in bibliometric analysis techniques [14,65,66,67]. This is due to its significance in a variety of fields, including shared word analysis and information consultation, and its function as a filter in research searches. The resulting keyword network provides insight into the relationships between research topics, highlighting emerging themes such as sustainable food packaging, biodegradable materials, and the application of pectin in edible films. This analysis shows the focus of research on eco-friendly packaging innovations and the functional properties of pectin, especially its antimicrobial attributes and mechanical durability. The keyword occurrence network of the selected articles is shown in Figure 4.

Figure 3a shows that the author keywords with the highest frequency, represented by the largest circles, are “pectin”, “food packaging”, “edible films”, “active packaging”, and “biopolymers”. The keywords were categorized into eight clusters based on bibliometric mapping generated by VOSviewer software. The cluster shown in red has the highest number of items, covering terms mostly centered on pectin prevention. These keywords include chitosan, Pickering emulsion, controlled release, film, and high- and low-methoxyl pectin. The purple and light blue clusters grouped keywords related to their application in food packaging (polyvinyl alcohol, pectin film, polyphenols, and mechanical properties) and their application in edible films (shelf-life, biopolymers, and composite films). The green cluster, which contains 10 items, groups keywords that focus on active packaging that preserves or extends the shelf life of products (antimicrobial activity, antioxidant activity, antimicrobial, preservation, nanocomposite, and nanoemulsion). In addition, the dark blue cluster focuses on sustainability aspects (biodegradability, biocomposites, bioplastics, circular economy, and byproducts). Finally, the yellow and orange clusters focus on the general packaging characteristics (films, coatings, tensile strength, and water vapor permeability) and polymer sources derived from polysaccharides (essential oils, packaging films, and biodegradability). From the data in Figure 3a, it can be said that pectin-based natural polymers are promising for applications in food packaging because of their good mechanical, physical, and biodegradable properties and characteristics. This has been confirmed by previous research [68], which assessed the best performance of pectin-based edible films in terms of thermal, mechanical, and gas barrier properties. In addition, the blending of pectin and pullulan can provide high strength and thermal stability to the resulting edible film [60].

In addition to explaining publication trends, Figure 3b shows keywords based on the time revolution. The keywords that are orange–red in color and have large circles are the keywords that have been widely researched by scientists. It can be seen that mechanical properties often associated with tensile strength and water vapor permeability have long been important indicators in the manufacture of pectin-based packaging. Films with high water vapor permeability and low tensile strength severely limit their applications in the food packaging industry [69,70]. Keywords such as antimicrobial activity, antioxidant activity, preservation, shelf life, sustainability, and biodegradability are currently being discussed. These keywords are currently the top of mind because of the demand for plastics that not only have good properties and characteristics but are also environmentally friendly, sustainable, and can maintain the quality and safety of the packaged product. Recent research has added antioxidant compounds [10,46], nanoparticles [71,72], and essential oil [73,74,75,76] to a pectin-based packaging matrix to maintain quality and extend product shelf life.

## 5. Application of Pectin-Based Materials in Active and Intelligent Food Packaging

Although pectin can form packaging films with high mechanical properties and barriers, the functionalities of pectin-based films still need to be improved by adding more bioactive compounds to increase their capability to protect food products and prolong their shelf life. The addition of active compounds to packaging improves the functional properties and extends the shelf life of products. The use of extracts derived from plants, animals, and microorganisms has been recognized as a valuable component that improves the functional properties of pectin-based films and coatings [77,78]. In addition, chemical compounds in the form of nanoparticles are frequently used as antimicrobial agents in food packaging [79].

Table 1 provides an overview of pectin-based active packaging applications in various food products. Diverse pectin sources, such as citrus, watermelon, and broccoli leaf pectin, are combined with different film components and active agents to produce packaging materials with enhanced functional properties. For example, the use of polydopamine-coated lignin nanoparticles (LNP@PDA) in citrus pectin-based composite films not only improves the mechanical strength and water resistance but also provides UV protection and high biological activity [80]. These results successfully extended the shelf life of bananas and milk [80]. In addition, multi-active films containing chitosan, epigallocatechin gallate (EGCG), and natamycin (NATA) showed significant improvements in UV protection, mechanical properties, and gas barrier properties, which are effective in maintaining strawberry freshness [81]. Pectin from watermelon rind combined with potato starch, TiO_2_ nanoparticles, and Lycium barbarum leaf flavonoids produced a composite film capable of improving mechanical strength, thermal resistance, and antimicrobial properties, which was effective in inhibiting microbial growth and chemical damage to Tan goat meat [82].

Pectin can be combined with other polymers to improve the mechanical, physical, and barrier properties, ultimately extending the shelf life and improving the quality of food products. For example, persimmon pectin mixed with sodium alginate, guar gum, and baobab seed oil, as well as broccoli leaf pectin combined with tapioca starch and broccoli leaf polyphenols (BLPs), showed significant improvements in mechanical strength, water resistance, and biological activity, which were effective in extending the shelf life of mushrooms and chilled mutton [30,83]. In addition, citrus pectin-based films with carbon quantum dots from garlic showed enhanced antioxidant and antibacterial activities as well as better mechanical properties, which successfully extended the shelf life of strawberries [84]. The combination of pectin with polyhydroxyalkanoate (PHA) and coffee ground extract showed effective water barrier ability and antimicrobial activity in preserving mashed carrots [85]. These studies confirm that pectin, with various modifications and combinations, provides a promising solution for safer and more durable food packaging, improving the quality and extending the shelf life of food products.

**Table 1:** Application of pectin-based active packaging in enhancing the shelf life of food products.

Pectin Source	Film/Coating Components	Active Agents	Food Products	Improved Film Properties	Shelf Life of Product Improved	Ref.
Citrus pectin	Polydopamine-coated lignin nanoparticles (LNP@PDA)	Antioxidants, antibacterial agents, UV-blocking agents	Bananas, milk	TS: 35.76 Mpa, WCA: 92.42°, UV blocking: 100% (UVA, UVB, UVC)	7 days (control 3–4 days)	[80]
Citrus pectin	Chitosan, pectin, epigallocatechin gallate (EGCG), natamycin (NATA)	Antioxidants (EGCG), antifungal (NATA)	Strawberries	TS: 71.64 Mpa, UV bloking: <1% (200–350 nm), WPV: 0.69 × 10^−13^ kg⋅m^−1^⋅s^−1^⋅Pa^−1^	8 days (control 1–2 days)	[81]
Watermelon peel pectin	Potato starch, TiO_2_ nanoparticles, Lycium barbarum leaf flavonoids (MLFs)	Antioxidants (MLFs), antimicrobial (nano-TiO_2_)	Tan mutton	TS: 45.9% improvement, WPV: decreased by 18%, thermal stability: maintained up to 220 °C, antioxidant activity: significant reduction in lipid oxidation	15 days (control 9 days)	[82]
Persimmon pectin	Sodium alginate, guar gum, β-Cyclodextrin, baobab seed oil	Antioxidants (*Lycium ruthenicum extract*), antibacterial agents (*Silver nanoparticles*)	Mushrooms	TS: 15.87 MPa, WVP: 4.82 g/m^2^.h.kPa, WCA: 91.23°, antioxidant activity: 88.26%	30 days	[71]
Broccoli leaf pectin	Tapioca starch, broccoli leaf polyphenols (BLPs)	Antioxidants (BLPs)	Chilled mutton	TS: 9.34 MPa, EB: 10.91%, WVP: 2.61 g⋅mm/(m^2^⋅h⋅Pa), antioxidant activity: 88.24% DPPH scavenging rate	12 days	[83]
Citrus peel pectin	Garlic-derived carbon quantum dots (CDs)	Antioxidants (CDs), antibacterial agents (CDs)	Strawberries	TS: 6.96 MPa, EB: 36.85%, WVP: 1.057 × 10^−^⁹ g m^−1^ h^−1^ Pa^−1^, antioxidant activity: 50% scavenging rate	5 days	[84]
Citrus pectin	Chitosan, jujube seed powder	Antioxidants (polyphenols), antimicrobial agents	Grapes	TS: 0.8375 MPa, EB: 38.25%, WVP: 33.71 × 10^−^⁹ g cm^−1^ s^−1^ Pa^−1^, antioxidant activity: 98.02% DPPH scavenging rate	10 days	[86]
Citrus peel pectin	Sodium alginate, calcium chloride, glycerol	Cinnamic acid	Fresh beef	TS: 0.124 MPa, EB: 13.88%, WVP: 2.915 × 10^−^⁹ g m/m^2^ s Pa, antibacterial activity: 84.09% reduction in bacterial load	5 days	[87]
Citrus peel powder (orange, lemon, pomelo, mandarin)	Sodium alginate, glycerol	Polyphenols, carotenoids, essential oils	Corn oil	TS: 8.26–9.14 MPa, EB: 8.05–17.18%, WVP: 1.34–1.92 × 10^−1^⁰ g m^−1^ s^−1^ Pa^−1^, antioxidant activity: strong (based on DPPH assay)	15 days	[88]
Pectin	Ovalbumin (OVA), chitosan (CS), gallic acid (GA)	Antioxidants (GA), antibacterial agents (GA)	Salmon fillets	TS: 15.97 MPa, EB: 7.29%, WVP: low, antibacterial activity: effective against *E. coli* and *M. morganii*	Extended by 3 days	[89]
Citrus pectin	Polyhydroxyalkanoates (PHAs), spent coffee ground (SCG) extract	Antioxidants (chlorogenic acid), antimicrobial agents (caffeoylquinic acid isomers)	Mashed carrots	TS: 9.1 MPa, EB: 16.1%, WVP: reduced significantly, antioxidant and antimicrobial activity: high	Extended by 3 days	[85]
Pectin	Chitosan (CS), calcium propionate (CP), curcumin-β-cyclodextrin (Cur-β-CD)	Antioxidants (Cur), antibacterial agents (CP, Cur)	Pork	TS: moderate, WVP: 4.55 × 10^−11^ g⋅(m⋅s⋅Pa)^−1^, antibacterial activity: 79.41% against *E. coli*, 83.82% against *S. aureus*		[9]
Watermelon peel pectin	Polyphenols from watermelon peel (WME), glycerol	Antioxidants (polyphenols), antimicrobial agents (polyphenols)	Chilled mutton	TS: 9.1 MPa, EB: 16.1%, WVP: reduced significantly, antioxidant and antimicrobial activity: high	Extended by 35 days	[90]
Pectin	Chitosan (CS), Tween-80	Antioxidants (α-Tocopherol)	Fatty food simulant	TS: 16.64 MPa, water uptake: 163.91%, antioxidant activity: up to 90.60% DPPH scavenging rate	Sustained release over 10 days	[91]
Grapefruit peel pectin (GFPec)	Grapefruit peel methanolic extract (GFPE), maltodextrin-encapsulated lemon peel extract (MD-LPE), PEG400	Antioxidants (GFPE, MD-LPE), antimicrobial agents (GFPE, MD-LPE)	Cherry tomatoes	TS: 15.09 MPa, EB: 19.12%, WVP: reduced significantly, antioxidant and antimicrobial activity: high	Extended by 6 days	[92]
Pectin	Chitosan, gelatin, glycerol, Tween 80	Lemongrass essential oil (LEO), ZnO, Zn(CH_3_COO)_2_⋅2H_2_O	Raspberries	TS: 16.87–21.78 MPa, EB: 48.69–73.04%, WVP: moderate, antimicrobial activity: high against *S. aureus* and *E. coli*	Extended by 8 days	[93]
Passion fruit peel pectin	Corn starch, glycerol, turmeric essential oil (TEO)	Antioxidants (TEO), antimicrobial agents (TEO)	Sliced bread	TS: 10.94 MPa, EB: 61.85%, WVP: 5.11 × 10^−^⁷ g⋅h^−1^⋅m^−1^⋅Pa^−1^,	No fungal contamination for 9 weeks	[94]

TS: tensile strength; EB: elongation at break; and WVP: water vapor permeability.

Intelligent packaging is currently in the spotlight. Interest in intelligent packaging is a sign of rising customer awareness and a growing sense of accountability among supply chain managers and producers. The ever-evolving challenges in the contemporary era and the search for better packaging solutions can be directly linked to this concept. The advent of new technologies not only strengthens control over efficient chain management but also helps prevent significant health problems from arising. Intelligent packaging usually illustrates its essence by reflecting sufficient intelligence to capture the preferences of end consumers [15,79,95]. In other words, a packaging system can be considered “intelligent” if it possesses the capacity to identify alterations in its surroundings and can carry out sophisticated operations such as identification, tracking, documentation, interaction, and computation to prolong its shelf life, thereby furnishing data and warnings [96,97,98].

Table 2 shows various applications of pectin-based intelligent packaging used to detect the freshness and quality of various food products. This intelligent packaging utilizes the color changes produced by the interaction between pectin and natural colorants in response to changes in pH and ammonia, which are key indicators of food freshness. In shrimp products, several studies have shown that pectin-based films combined with anthocyanins from different sources are effective in providing a visual indication of product freshness. The combination of gelatin, pectin, and glycerol with anthocyanins from pistachio shells resulted in a color change from pink/brown to yellow/brown, indicating the freshness and deterioration of shrimp at various pH levels [99]. Meanwhile, films combining pectin and chitosan nanostructures with anthocyanins from sumac showed rapid color changes from red to olive green within 5 min and from red to green after 48 h of storage, indicating high sensitivity to ammonia [100]. Another combination, pectin with sodium alginate and CNCs and anthocyanins from red cabbage, showed a color change from purple to dark green or greenish yellow after 72 h at 25 °C, with a slower change at 4 °C, providing an accurate indication of the freshness of shrimp under various storage conditions [20].

Based on the data in Table 2, most intelligent packaging systems use a pH-based sensing mechanism, with color change as the visible reaction. This approach is often used due to its simplicity and effectiveness in monitoring changes in food quality and spoilage. However, intelligent packaging technologies can include broader functions beyond pH-based color changes, such as temperature sensors, gas concentration, and microbial detection, which provide more comprehensive monitoring. For example, Choi and Han [103] showed that gas-based intelligent packaging can detect changes in CO_2_ levels inside the package. As CO_2_ increases, there is a pH change in the NaCas–pectin solution that causes a change in transparency, signaling the fermentation stage and food quality. Although most of the current pectin-based intelligent packaging systems focus on pH and color change detection, the potential for expanding these intelligent features is still enormous and requires further research. Some similar applications using other polymers have incorporated functions such as temperature-sensitive labels (e.g., thermochromic ink-based packaging) [106], gas detection sensors (e.g., ethylene sensors for monitoring fruit ripening) [107], and RFID-enabled systems for tracking and interaction [108]. Overall, pectin-based intelligent packaging has great potential for improving the safety and quality of food products.

Pectin-based films, as in Table 1 and Table 2, generally have moderate tensile strength, ranging from 6.96 MPa to 9.34 MPa. Meanwhile, when compared to protein-based films, gelatin in particular, can have a wider range of tensile strengths, from 5.8 MPa to 15.4 MPa, depending on the additives [109]. This suggests that protein-based films, particularly gelatin, can achieve higher strength under certain conditions. In terms of elongation to break, pectin-based films show more limited flexibility with a range of 10% to 36.85%, whereas protein-based films, especially gelatin, show a much wider range. Gelatin, for example, can stretch up to 471% [109], which indicates that protein-based films have a much higher degree of flexibility. This makes them more suitable for applications that require high elasticity. Although pectin-based films still need to be improved in terms of their flexibility and mechanical strength, the use of composites with other materials can help strengthen their mechanical properties, such as the addition of chitosan increasing the tensile strength of pectin films to 71.64 Mpa [81]. The combination of pectin with various polymers, natural colorants, and other additives not only improves the mechanical and barrier properties of the film but can also extend the shelf life of the product and provide an effective detection function. This innovation can be a more sustainable and environmentally friendly solution than conventional plastics, supporting global efforts to reduce plastic waste and improve food safety.

## 6. Limitations of Pectin-Based Packaging

Pectin-based packaging has various benefits, including biodegradability, renewability, non-toxicity, gas barrier capability, high mechanical properties, superior rheological properties, cost-effectiveness, and strong film-forming capacity [110,111]. Despite the considerable advantages of pectin-based packaging in active and intelligent packaging applications, certain obstacles need to be overcome to achieve wider implementation. Significant obstacles still exist, including inefficiency in preventing moisture transfer, inadequate mechanical characteristics, brittleness, low thermal stability, and excessive water solubility. For example, films composed solely of pectin exhibit significant susceptibility to tearing and cannot withstand substantial mechanical stress. These constraints hinder their use in packaging and require strong load or physical stress resistance. To overcome these problems, several attempts have been made to incorporate reinforcements, such as nanoparticles or other polymers, into the pectin matrix [112,113,114].

In addition, the incorporation of ionic metals, including calcium chloride, zinc chloride, and magnesium chloride ions, into the pectin matrix can improve the mechanical characteristics and water resistance of pectin films [104,115]. In addition, pectin sheets do not have good barrier characteristics against moisture and gas, thus reducing packaging efficacy. This is especially true for dry or fatty foodstuffs that require maximum protection from moisture or oxygen. Research results show that incorporating hydrophobic polymers or nanoparticles into pectin can improve its barrier characteristics [116]. The incorporation of bioactive substances, such as essential oils, nanoparticles, or indicator substances, is often necessary to improve the characteristics of pectin films used in active and intelligent packaging [117]. While incorporating such bioactive substances can improve film performance, this often results in stability issues when stored. For example, the unregulated liberation of active chemicals or the deterioration of bioactive substances caused by contact with moisture or oxygen can reduce the long-term efficacy of films. To address these issues, additional studies are needed to design approaches that can regulate the liberation of bioactive substances with greater accuracy, such as by encapsulation methods [118] or the use of protective coatings [119]. While improving the physical and mechanical characteristics of pectin films by integrating additional polymers, nanoparticles, or active substances is possible, this can also lead to higher production costs. Therefore, it will be difficult for pectin-based packaging films with improved physical and mechanical properties to compete with cheaper conventional plastics on a commercial scale.

## 7. Future Perspectives

Pectin has great potential as a base material for sustainable and environmentally friendly food packaging. The future of pectin research and its applications in food packaging includes several key inter-related areas. The development of more effective packaging formulations should continue by exploring the combination of pectin with other ingredients, such as nanoparticles, essential oils, and natural or synthetic polymers. These combinations are expected to improve the mechanical, thermal, and functional properties of packaging, thereby extending the shelf life of food via improved antimicrobial and antioxidant properties. Innovation in pectin-based intelligent packaging is key. More advanced sensor technology integrated into pectin packaging will enable real-time detection of changes in food quality without opening the packaging. This will improve consumers’ ability to directly assess food freshness, reduce food wastage, and increase their trust in packaged products.

The implementation of circular economy concepts in the production and use of pectin will strengthen its position as a sustainable packaging material. Collaboration between academia, industry, and the government is crucial to creating policies and best practices in waste management and pectin-based packaging production. This holistic approach ensures that the entire life cycle of pectin products supports global sustainability goals. The scale-up and commercialization of pectin products are also challenges that must be addressed to meet greater industrial demands. Research should be directed at economically and sustainably scaling up pectin packaging production. Economic feasibility studies and market analysis will help identify opportunities and challenges in the commercialization of pectin packaging, ensuring that production can be performed in a cost-effective and sustainable manner. By focusing on innovation, sustainability, and collaboration, pectin has the potential to become a key ingredient in future food packaging that is environmentally friendly and efficient. Further development in these areas will enable pectin to replace conventional plastics, provide safer and more durable packaging solutions, and support global efforts to reduce the environmental impacts of plastic waste.

## 8. Conclusions

Pectin is a natural polymer that has the potential to be used as a base material for biodegradable plastics because of its unique properties, such as biodegradability, biocompatibility, and the ability to form a sturdy film. In the food packaging industry, pectin shows significant promise as a primary material for active and intelligent packaging. Active packaging based on pectin incorporates antimicrobial and antioxidant compounds that help prolong the shelf life of food products by inhibiting bacterial growth and absorbing oxygen and water vapors. The use of nanoparticles and essential oils in the pectin matrix, for example, can improve mechanical strength and water resistance and provide UV protection and high biological activity. However, pectin-based intelligent packaging enables the monitoring of food quality without opening the package, with technology that can detect environmental changes and provide data and alerts on the freshness of packaged products. Various combinations of pectin with other components, such as natural and synthetic polymers, nanoparticles, and plant extracts, have shown promising results in improving functional properties and extending the shelf life of food products. For example, the combination of pectin with potato starch and TiO_2_ nanoparticles has been shown to improve the mechanical strength, thermal resistance, and antimicrobial properties of meat and fruits. 

Research trends on the use of pectin in food packaging have shown a significant increase over the past decade, reflecting the growing awareness of the need for environmentally friendly and sustainable packaging solutions. With the continuous development of new technologies and applications, pectin has great potential to reduce the dependence on conventional plastics and provide safer and more durable packaging solutions. Pectin offers innovative and environmentally friendly solutions for food packaging applications, providing effective protection against microbes and oxidation and improving the quality and safety of food products.

## Figures and Tables

**Table 2:** Application of pectin-based intelligent packaging in food packaging.

Film Components	Intelligent Agent	Sensing Type	Food Products	Improved Film Properties	Significant Findings	Ref.
Gelatin, pectin, glycerol	Pistachio peel (anthocyanin)	pH	Shrimp	TS: 0.7 MPa, EB: 56%, WVP: reduced from 2.81 to 2.74 g·s^−1^·Pa^−1^·m^−1^	Color changes from cherry/pink to yellow/brown indicating freshness and spoilage at different pH levels	[99]
Pectin, chitosan nanofiber	Sumac (anthocyanin)	Ammonia, pH	Shrimp	TS: 60 MPa, EB: 23.3%, WVP: 2.34 × 10^−11^ g/m^2^ s Pa	Color changes from reddish to olive color within 5 min, and from reddish to greenish after 48 h of storage	[100]
Pectin, sodium alginate, CNCs	Red cabbage (anthocyanin)	pH	Shrimp	TS: 17.19 MPa, EB: 39.18%, WVP: 7.10%	Color changes from lilac to dark green to greenish-yellow after 72 h at 25 °C; slower color change at 4 °C	[20]
Pectin, chitosan	Black rice (anthocyanin)	pH	Meat	TS: 57.3 MPa, elongation at break: 18.5%, WVP: 4.12 × 10^−11^ g/m^2^ s Pa	Changes color from red to blue as meat spoilage increases, showing the indicative effect on meat putrification	[101]
Pectin, chitosan, glycerol	Black rice (anthocyanin)	Ammonia, pH	Pork and Beef		Red to yellow-green color change indicating the spoilage of meat; sensitive to volatile basic nitrogen	
Pectin/anthocyanin	*Phaseolus vulgaris* (anthocyanin)	pH	Chicken meat	TS: 15 MPa, EB: 40%, WVP: moderate	A film that changed from pink to brownish with rising pH; the film has a strong ability to inhibit the bacterial growth of *E. coli* and *S. aureus*	[102]
Pectin, sodium caseinate	Sodium caseinate (*NaCas*)	Gas concentrations	Kimchi	Transparency change: from 80% to 30% upon exposure to CO_2_, pH-responsive: stable at pH 6.5, changes rapidly at pH 4.5	Strong correlations between kimchi quality, ripeness, and the indicator’s visible traits during storage	[103]
Pectin/carboxymethyl cellulose sodium/anthocyanins/metal ion	Blue honeysuckle berry	pH	Shrimp	TS: increased by 1.52 times, WVP: significantly reduced, thermal stability: improved with metal ion crosslinking	The film enhanced storage stability and antioxidant capacity, and effectively monitored shrimp freshness during storage	[104]
Pectin/starch/cyanidin/alizarin	Cyanidin/alizarin	pH	Pork	TS: increased by cassava starch addition, WVP: reduced significantly, pH-sensitive color change: red to blue–black with spoilage	The film showed strong sensitivity to volatile nitrogen, with visible color change over 10 days at 4 °C, enabling real-time spoilage monitoring	[105]

TS: tensile strength; EB: elongation at break; and WVP: water vapor permeability.

## Data Availability

Available data are presented in the manuscript.
